# Chronology of H3N2 human influenza virus surface glycoprotein adaptation from 1968 to 2019 reveals a surge of adaptation between 1997 and 2002

**DOI:** 10.1128/jvi.01329-25

**Published:** 2025-10-31

**Authors:** Hui Lei, Biying Xiao, Xia Lin, Zaolan Liang, Shiman Ling, Yaqin Bai, Vijaykrishna Dhanasekaran, Wenjun Song, Sook-San Wong, Mark Zanin

**Affiliations:** 1 School of Public Health, LKS Faculty of Medicine, The University of Hong Kong 25809, Hong Kong, Hong Kong; 2 Centre for Immunology & Infection, Hong Kong, Hong Kong; 3 State Key Laboratory of Respiratory Diseases, Guangzhou Medical University 26468, Guangzhou, China; 4 HKU-Pasteur Research Pole, LKS Faculty of Medicine, The University of Hong Kong 25809, Hong Kong, Hong Kong; 5 Guangzhou Laboratory, Guangzhou International Bio Island, Guangzhou, China; University Medical Center Freiburg, Freiburg, Germany

**Keywords:** influenza virus, H3N2, adaptation, receptor binding, pathogenicity

## Abstract

Subtype H3N2 influenza A viruses (IAVs), which emerged in 1968 to cause a pandemic, have shown continual circulation and adaptation that has necessitated frequent updates of candidate vaccine viruses. Here, we sought to determine how genetic changes in the hemagglutinin (HA) and neuraminidase of 21 antigenically distinct H3N2 IAVs isolated from 1968 to 2019 correlate with mammalian fitness and adaptation. We found a surge of adaptation between 1997 and 2002, resulting in the emergence of A/Fujian/411/2002 (H3N2) and poor vaccine efficacy, leading to an epidemic during the 2003–2004 season. This surge was characterized by a large reduction in binding to mammalian-type α2,6-linked sialic acids and increased infectivity and replication kinetics in humanized Madin-Darby canine kidney cells. HA glycosylation also increased most rapidly from 1968 to 2004 and then plateaued. Symptomatic infections were only evident in mice following inoculation with viruses isolated in the 1970s and A/Aichi/2/1968 (H3N2), which was the most pathogenic. More recent viruses did not cause any detectable symptoms, except for A/Sydney/5/1997 (H3N2), which caused some weight loss. The post-2002 shift to α2,6-linked sialic acid binding, coupled with reduced pathogenicity in mammalian models, underscores H3N2 adaptation to human circulation without affecting immunogenicity, which is a critical consideration for vaccine design. Overall, our data revealed that a surge of mammalian adaptation from 1997 to 2002 gave rise to A/Fujian/411/2002 (H3N2), with subsequent viruses showing more hallmarks of mammalian adaptation, such as increased binding to cells expressing α2,6-linked sialic acids and reduced mammalian pathogenicity.

**IMPORTANCE:**

The continued endemicity of subtype H3N2 influenza A viruses (IAVs) in humans necessitates an understanding of the continuing accumulation of mammalian adaptations to inform public health countermeasures. We used a combined approach of studying the genetic, antigenic, and pathogenic adaptations of the surface glycoproteins of 21 H3N2 IAVs representing distinct antigenic groups from 1968 to 2019. We observed a loss of mammalian pathogenicity within 10 years of human circulation and a surge of adaptation between 1997 and 2002 that gave rise to A/Fujian/411/2002 (H3N2), which was a poor match for vaccine viruses. This surge was characterized by large shifts in glycan binding preferences, antigenicity, and genetic evolutionary distances. Overall, our study reveals novel insights into the chronology of the mammalian adaptation of H3N2 IAVs.

## INTRODUCTION

Human influenza A H3N2 viruses have circulated continuously since their emergence in 1968, providing a unique lens into the long-term evolution of a major respiratory pathogen. The H3N2 subtype triggered the “Hong Kong flu” pandemic of 1968 to 1970, causing over a million deaths worldwide (1, 2). These viruses originated through reassortment, combining avian-derived hemagglutinin (HA) and polymerase basic 1 (PB1) genes with six genes from the previously circulating H2N2 viruses, including neuraminidase (NA) (3). A key adaptation was the early gain of binding preference for α2,6-linked sialic acids, a prerequisite for efficient human-to-human transmission. Since then, H3N2 has remained a leading cause of seasonal influenza morbidity and mortality worldwide (4).

Compared to other human influenza viruses, such as H1N1 (1918–2009), H1N1pdm09 (2009–present), H2N2 (1957–1968), and influenza B, H3N2 exhibits exceptionally rapid antigenic evolution (5–7). Antigenic variants emerge every 2 to 5 years through HA substitutions that escape pre-existing immunity, necessitating frequent updates to the H3N2 vaccine component, 37 times to date (8). This antigenic instability underscores the public health risks posed by this subtype.

H3N2 adaptation has involved a gradual shift in receptor specificity and binding avidity. Between 1968 and the early 1990s, the virus transitioned from dual α2,3/α2,6 binding to exclusive α2,6 recognition (9, 10), with biophysical analysis showing a gradual evolution from mixed α2,3/α2,6 binding to α2,6 specificity by 1979, followed by a decline in binding avidity after 1992 (11). By 1992, clinical isolates no longer agglutinated chicken red blood cells, an indicator of diminished avian receptor binding (12). These changes correlate with reduced infectivity in standard Madin-Darby canine kidney (MDCK) cells, favoring infection in MDCK cells expressing the cDNA of human 2,6-sialyltransferase (SIAT1) (MDCK-SIAT1 cells) engineered to express human-type receptors (13). Concurrently, H3N2 viruses evolved to preferentially bind elongated α2,6-linked sialic acids, likely under immune pressure (14–16). These receptor adaptations coincided with increasing glycosylation near the HA receptor-binding site, which both attenuates receptor engagement and masks antigenic epitopes (17, 18). Given the overlap of receptor-binding and antigenic sites (A, B, D), mutations conferring immune escape often simultaneously affect receptor interactions (13, 15, 19, 20).

H3N2 antigenic drift has practical implications for vaccine efficacy (VE). Notably, VE against H3N2 viruses has been suboptimal in multiple seasons, at only 22%–36% in the USA during the 2016–2017, 2017–2018, and 2021–2022 influenza seasons, despite antigenic similarity between vaccine and circulating strains (21–23). While HA is the focus, NA immunity is elicited by natural infection and vaccination, although the NA content of seasonal influenza vaccines is not standardized (24–26). NA antigenic drift has also been observed, exemplified by the emergence of S245N and S247T in 2016, which introduced an *N*-linked glycosylation site in a conserved epitope overlapping the active site (27, 28). This led to reduced neuraminidase inhibition (NAI) titers, reduced binding of monoclonal antibodies raised against earlier strains, and reduced protection afforded by immunity generated by earlier viruses lacking this glycosylation site *in vivo* (27, 28). Furthermore, reduced VE may be further compounded by adaptive mutations introduced during egg-based vaccine production (29, 30).

Poor VE was particularly evident upon the emergence of the antigenically variant strain A/Fujian/411/2002 (H3N2), which was a poor antigenic match to the 2002–2003 vaccine strain A/Moscow/10/1999 (H3N2) (31, 32). This led to an epidemic during the 2003–2004 influenza season (6, 33, 34).

While specific genetic or antigenic shifts in H3N2 strains have been documented, an integrative, longitudinal approach to understanding how key phenotypic traits, namely receptor binding, antigenicity, replication, and pathogenicity, co-evolve over time is needed. The persistent success of H3N2 viruses likely reflects the coordinated evolution of these traits. In this study, we reconstructed 5 decades of H3N2 evolution (1968–2019) using 21 recombinant viruses representing major antigenic clusters and vaccine strains. We assessed receptor specificity, glycosylation, hemagglutination, replication in mammalian cells, and pathogenicity in mice. These traits were selected based on their established roles in host adaptation, immune evasion, and disease severity. Our findings reveal a phased trajectory of adaptation, including early loss of avian features and a major evolutionary inflection between 1997 and 2002, marked by shifts in receptor binding and cell tropism. This longitudinal analysis provides an integrated perspective on the phenotypic strategies that have enabled H3N2 viruses to persist and adapt in humans over 5 decades.

## MATERIALS AND METHODS

### Virus selection, generation, and sequencing

Twenty-one human H3N2 viruses isolated between 1968 and 2019 were selected for this study (Table 1). Viruses from 1968 to 2003 were chosen to represent defined antigenic clusters, while those from 2004 to 2019 corresponded to WHO candidate vaccine viruses (5, 8). Where possible, the HA and NA sequences from cell-grown viruses were used. However, the HA and NA sequences of A/Aichi/2/1968 (H3N2), A/England/42/1972 (H3N2), A/Victoria/3/1975 (H3N2), A/Sichuan/2/1987 (H3N2), and A/Wuhan/359/1995 (H3N2) were obtained from egg-passaged viruses, as sequences from cell-passaged viruses were not available.

**TABLE 1:** Subtype H3N2 influenza A viruses used in this study in chronological order

Group	Virus strain	Abbreviation	GenBank ID of hemagglutinin	GenBank ID of neuraminidase
Early Group	A/Aichi/2/1968 (H3N2)^*a*^	AI68	CY121117.1	CY121119.1
	A/England/42/1972 (H3N2)^*a*^	EN72	CY121157.1	CY121159.1
	A/Victoria/3/1975 (H3N2)^*a*^	VI75	CY121197.1	CY121199.1
	A/Texas/1/1977 (H3N2)	TX77	CY113261.1	CY120994.1
	A/Bangkok/1/1979 (H3N2)	BK79	CY114429.1	CY114431.1
	A/Sichuan/2/1987 (H3N2)^*a*^	SI87	CY121293.1	CY112398.1
	A/Beijing/352/1989 (H3N2)	BE89	CY112404.1	CY112406.1
	A/Beijing/32/1992 (H3N2)	BE92	CY113677.1	CY113679.1
	A/Wuhan/359/1995 (H3N2)^*a*^	WU95	CY112821.1	CY112823.1
	A/Sydney/5/1997 (H3N2)	SY97	CY112885.1	CY112887.1
Recent Group	A/Fujian/411/2002 (H3N2)	FU02	CY112933.1	CY112935.1
	A/California/7/2004 (H3N2)	CA04	CY114373.1	CY114375.1
	A/Wisconsin/67/2005 (H3N2)	WI05	CY163648.1	CY163906.1
	A/Perth/16/2009 (H3N2)	PE09	KM821346.1	CY081429.1
	A/Victoria/361/2011 (H3N2)	VI11	KM821347.1	KJ942682.1
	A/Switzerland/9715293/2013 (H3N2)	SWZ13	MW298184.1	MW298251.1
	A/Hong Kong/4801/2014 (H3N2)	HK14	MW298183.1	OQ350820.1
	A/Singapore/INFIMH-16-0019/2016 (H3N2)	SG16	MW298182.1	OQ350097.1
	A/Kansas/14/2017 (H3N2)	KA17	OR552530.1	MG974452.1
	A/Hong Kong/2671/2019 (H3N2)	HK19	OR533780.1	OR533782.1
	A/South Australia/34/2019 (H3N2)	SA19	EPI1387331	EPI1387330

^*a*^ Sequences were obtained from egg-grown viruses.

pHW2000 plasmids encoding the HA or NA genes listed in Table 1 were synthesized (Sangon Biotech) and used to generate recombinant viruses by eight-plasmid reverse genetics using the HA and NA genes from each strain with the six internal genes of A/Puerto Rico/8/1934 (H1N1) (35). Viruses were propagated in the allantoic cavity of 9-day-old specific-pathogen-free embryonated chicken eggs, as these viruses replicated to high titers in this background. We confirmed that no mutations were introduced following propagation in chicken eggs by Sanger sequencing using strain-specific primers and comparison with published sequences (Table 2). Viral RNA was extracted using the FastPure Viral DNA/RNA Mini Kit (Vazyme) and reverse transcribed using HiScript II One Step RT-PCR Kit (Vazyme).

**TABLE 2:** Sequences of primers used to amplify viral gene segments

Primer name	Primer sequence (5′→3′)
Ba-PB2-1	TATTGGTCTCAGGGAGCGAAAGCAGGTC
Ba-PB2-2341R	ATATGGTCTCGTATTAGTAGAAACAAGGTCGTTT
Bm-PB1-1	TATTCGTCTCAGGGAGCGAAAGCAGGCA
Bm-PB1-2341R	ATATCGTCTCGTATTAGTAGAAACAAGGCATTT
Bm-PA-1	TATTCGTCTCAGGGAGCGAAAGCAGGTAC
Bm-PA-2233R	ATATCGTCTCGTATTAGTAGAAACAAGGTACTT
Bm-HA-1	TATTCGTCTCAGGGAGCAAAAGCAGGGG
Bm-NP-1	TATTCGTCTCAGGGAGCAAAAGCAGGGTA
Bm-NP-1565R	ATATCGTCTCGTATTAGTAGAAACAAGGGTATTTTTC
Ba-NA-1	TATTGGTCTCAGGGAGCAAAAGCAGGAGT
Ba-NA-1413R	ATATGGTCTCGTATTAGTAGAAACAAGGAGTTTTTT
Bm-M-1	TATTCGTCTCAGGGAGCAAAAGCAGGTAG
Bm-M-1027R	ATATCGTCTCGTATTAGTAGAAACAAGGTAGTTTTT
Bm-NS-1	TATTCGTCTCAGGGAGCAAAAGCAGGGTG
Bm-NS-890R	ATATCGTCTCGTATTAGTAGAAACAAGGGTGTTTT

### Genetic analysis and glycosylation site prediction

Maximum likelihood phylogenetic trees of HA and NA amino acid sequences were constructed using IQ-TREE (version 2.0.3), with final model parameter optimization, and visualized by FigTree (version 1.4.4). The clades were colored according to the antigenic map of subtype H3N2 influenza A viruses (IAVs) and rooted to A/Aichi/2/1968 (H3N2) (5). Genetic distances were calculated using MEGA 11 using amino acid sequences, the p-distance model, and the maximum likelihood method and 1,000 bootstrap replicates. Distances were visualized in GraphPad Prism 9.1.1. *N*-linked glycosylation sites in HA and NA were predicted using NetNGlyc 1.0 (https://services.healthtech.dtu.dk/services/NetNGlyc-1.0/).

### Cell culture

MDCK cells were maintained in minimal essential medium (MEM) supplemented with 10% fetal bovine serum (FBS), 100 units/mL penicillin, 100 µg/mL streptomycin, 1% L-glutamine, and 1% vitamins solution. Humanized MDCK (hCK) cells were maintained in MEM supplemented with 5% FBS, MEM amino acids, MEM vitamins solution, 1% L-glutamine, 100 units/mL penicillin, 100 µg/mL streptomycin, 10 µg/mL blasticidin, and 2 µg/mL of puromycin. Cells were incubated at 37°C in a humidified atmosphere with 5% CO_2_. Infection media contained MEM with 0.3% bovine serum albumin (BSA), 100 units/mL penicillin, 100 µg/mL streptomycin, and 1% L-glutamine. Human embryonic kidney 293T cells were cultured in Dulbecco’s modified Eagle medium containing 10% FBS, 100 units/mL penicillin, 100 µg/mL streptomycin, 1% L-glutamine, and 1% vitamins.

### Virus titration and replication kinetics

Virus titers were determined by plaque assay in MDCK or hCK cells. Replication kinetics were assessed in two independent experiments, each with four replicates. Cells were infected at a multiplicity of infection of 0.01, and culture supernatants were harvested at designated time points and stored at −80°C. Titers were quantified by tissue culture infectious dose 50% in MDCK or hCK cells using the Reed and Muench method (36).

### Hemagglutination and hemagglutination inhibition (HAI) assays

Hemagglutination (HA) assays were performed using 0.5% (vol/vol) chicken red blood cells (cRBCs) and 0.75% (vol/vol) guinea pig RBCs (gRBCs) (37). HA titers were read after incubation at room temperature for 30 min (cRBCs) or 60 min (gRBCs) and expressed as the reciprocal of the highest virus dilution showing complete agglutination. Samples below the detection limit (1:10) were assigned a titer of five.

HAI assays were conducted using receptor-destroying enzyme (Accurate Chemical)-treated serum in 96-well plates. Serial serum dilutions were incubated with four HA units of virus for 1 hour at room temperature, followed by the addition of 0.75% gRBCs. HAI titers were defined as the highest serum dilution that completely inhibited hemagglutination.

### Receptor-binding assay

Receptor-binding specificities were determined by solid-phase enzyme-linked receptor-binding assay as previously described (38). Virus stocks were prepared by passage in eggs followed by purification and concentration over a cushion of 25% sucrose in STE buffer (0.1M NaCl, 10 mM Tris-HCl, 1 mM EDTA, pH 8.0) and ultracentrifugation at 25,000 rpm for 1 hour at 4°C. The biotinylated sialic acid receptor analogs used were; 3´-sialyllactose (3´SL, Neu5Acα2-3Galβ1-4Glcβ-PAA-biotin), 6´-sialyllactose (6´SL, Neu5Acα2-6Galβ1-4Glcβ–PAA-biotin), 3´-sialyl-N-acetyllactosamine (3´SLN, Neu5Acα2-3Galβ1-4GlcNAc) and 6´-sialyllactosamine (6´SLN, Neu5Acα2-6Galβ1-4GlcNAcβ-PAA-biotin) (GlycoNZ).

Polyvinyl chloride enzyme immunoassay (EIA) microplates (Corning) were coated with 200 µL/well of bovine fetuin in phosphate-buffered saline (PBS) (10 µg/mL) overnight and then washed with distilled water. Plates were then blocked with 200 µL/well PBS containing 5% BSA at room temperature for 1 hour. Viruses, normalized by HA titer, were diluted to 128 HA units in 50 µL PBS and added to the fetuin-coated plates for incubation at 4°C overnight, followed by washing with an ice-cold PBS washing buffer. Serial twofold dilutions of respective sialylglycopolymers in reaction buffer (0.02% BSA, 0.01% Tween 80, 1 µM zanamivir in PBS) were then added at 100 µL/well and incubated at 4°C for 2 hours. Plates were then washed five times with PBS containing 0.05% Tween-20, then 100 µL of streptavidin-peroxidase conjugates (Invitrogen) diluted 1/2,000 in reaction buffer were added to each well and incubated for 1 hour at 4°C. Plates were then incubated with 3,3´,5,5´-Tetramethylbenzidine (Sigma) for 10 min at room temperature for color formation, and reactions were then stopped using 50 mM HCl. Optical densities were measured at 450 nm in a Synergy 2 multi-mode microplate reader (BioTek Instruments). The apparent association constant (*K*_ass_) for each virus-receptor interaction was calculated using Scatchard plot analysis.

### Enzyme-linked lectin assay

NAI titers in mouse serum were determined using enzyme-linked lectin assays (ELLA). Recombinant viruses were incubated in fetuin-coated 96-well plates at 37°C for 18 hours. Concentration of viruses used in ELLA was standardized using a twofold dilution curve. The virus dilution used was within the linear portion of the titration curve, with OD_450nm_ equal to 95% of the maximum achievable OD and at least 10 times higher than the background (no virus) control. Plates were then washed and incubated with peanut agglutinin-horseradish peroxidase(PNA-HRP) for 2 hours at room temperature. After development with O-phenylenediamine dihydrochloride, absorbance was measured at 490 nm. For NAI titration, serum samples were serially diluted from 1:10, incubated with virus, and added to fetuin-coated plates overnight at 37°C. The highest serum dilution yielding ≥50% inhibition was recorded as the NAI titer.

### Neuraminidase activity

Neuraminidase enzymatic activity was measured using the fluorogenic substrate 2´-(4-methylumbelliferyl)-α-_D_-*N*-acetylneuraminic acid (MUNANA; Sigma) (39, 40). Viruses were normalized by HA titer, serially diluted, and incubated with 100 µM MUNANA at 37°C for 30 min. Reactions were stopped with a solution of 25% (vol/vol) ethanol and 12.5% (vol/vol) glycine (Fisher Scientific) in distilled water, and fluorescence was measured at excitation/emission wavelengths of 360/460 nm. Data represent the mean of three independent experiments.

### Mouse experiments

Female BALB/cJ mice (6 weeks old; Zhejiang Weitong Lihua Experimental Animal Technology Co.) were anesthetized with isoflurane and intranasally inoculated with 10^3^, 10^4^, or 10^5^ PFU) of virus in 30 µL PBS, while control mice received PBS alone. Animals were monitored at least daily for 14 days for weight loss and clinical signs and euthanized upon reaching humane endpoints (≥30% wt loss or severe illness). Sera were collected 3 days pre- and 28 days post-inoculation.

### Statistical analyses

Statistical analyses were performed using GraphPad Prism 9.1.1 (GraphPad Software Inc.). Comparisons between two groups were made using unpaired *t*-tests; comparisons among multiple groups were conducted using one-way analysis of variance. Survival curves were compared using the Kaplan-Meier method with log-rank test. *P*-values <0.05 were considered statistically significant.

## RESULTS

### Emergence of A/Fujian/411/2002 (H3N2) was associated with progressive accumulation of predicted glycosylation sites on HA, but not NA, and greater specificity for α2,6-linked sialic acids

**Fig 1:**
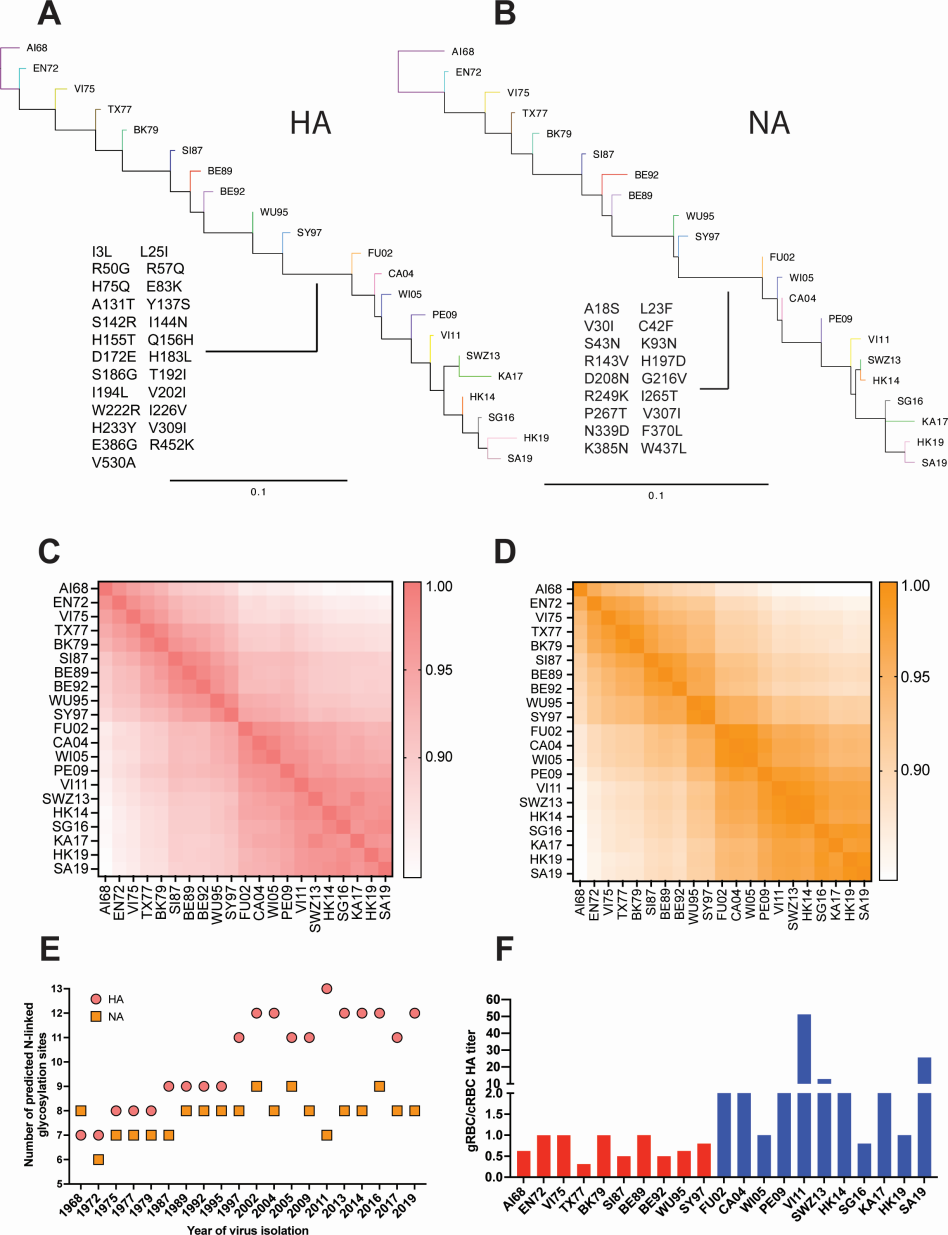
Temporal dynamics of the genetic evolution, predicted *N*-linked glycosylation sites, and sialic acid binding preferences of the surface glycoproteins of subtype H3N2 influenza A viruses circulating in humans between 1968 and 2019. Maximum likelihood phylogenetic trees of HA (**A**) and NA (**B**) showing the amino acids marking the transition from SY97 to FU02. Evolutionary analyses of the HA (**C**) and NA (**D**) genes were performed in MEGA v.11, with evolutionary distances computed using the maximum likelihood method and 1,000 bootstrap replicates. (**E**) Numbers of predicted *N*-linked glycosylation sites in the HA and NA proteins of subtype H3N2 influenza A viruses over time. Data reflect the number of N-X-S/T sequons at surface-exposed positions on HA and NA across each strain analyzed (Tables 4 and 5). (**F**) Ratios of HA titers obtained using gRBCs over cRBCs of subtype H3N2 influenza A viruses. Virus strain name abbreviations can be found in Table 1.

**Fig 2:**
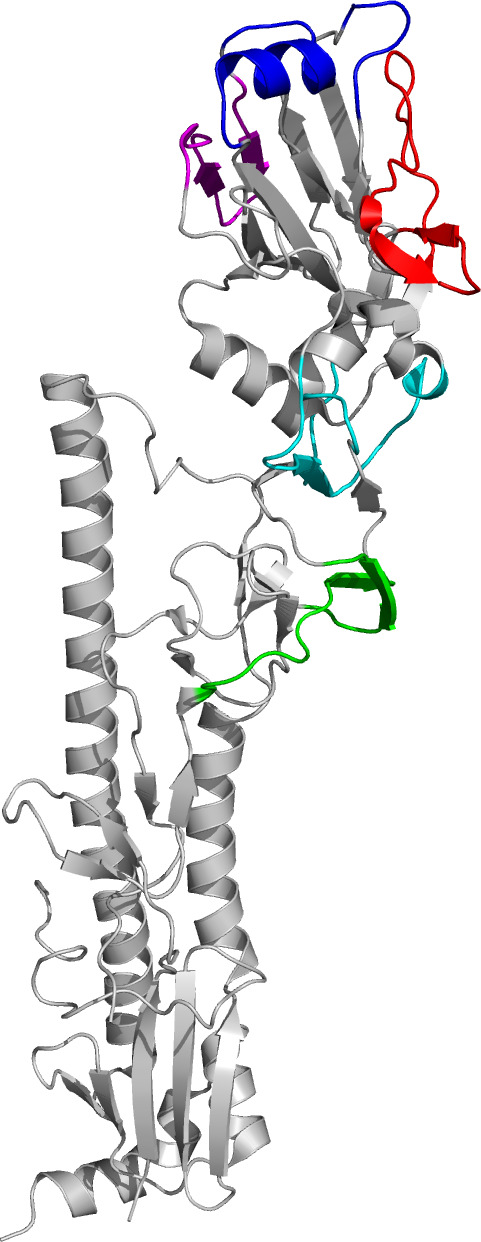
Cartoon of hemagglutinin showing locations of antigenic sites. Colors indicate antigenic sites. Red: antigenic site A. Blue: antigenic site B. Green: antigenic site C. Magenta: antigenic site D. Cyan: antigenic site E. H3 numbering of mature-form HA was used. Image was generated using The PyMOL Molecular Graphics System, version 3.0 Schrödinger, LLC, and structure 4O5N of the HA from A/Singapore/H2011.447/2011 (H3N2) (Protein Data Bank).

Homology modeling of genetic evolutionary distances between these 21 viruses spanning 1968 to 2019 revealed continual evolution, with the largest transition between SY97 and FU02, as shown previously (31, 32, 41). This has essentially given rise to two groups of IAVs: those isolated in 1968 to 1997 (Early Group) and in 2002 to 2019 (Recent Group) (Fig. 1A through D). The genetic distances of the HA and NA amino acid sequences between IAVs within these groups were of the ranges 83.2% to 98.7% and 85.6% to 99.4%, respectively. There were 12 stable amino acid changes differentiating the groups, at positions 25, 50, 57, 75, 137, 172, 192, 202, 222, 386, 452, and 530 (H3 numbering, Fig. 2; Table 3). Six of these amino acids are located in the HA globular head, with 57 and 75 in antigenic site E, 137 in the 130 ring of the receptor binding site (RBS) that is part of antigenic site A, 172 in antigenic site D, 192 in the 190 helix, and 222 in the 220 ring of the RBS (Fig. 2; Table 3) (42–44). Notably, FU02, the earliest Recent Group IAV, was the first with 156H and 225G, which are determinants of sialic acid binding specificity, with 225 additionally affecting HA stability and pH of activation (45–48).

**TABLE 3:** Common amino acid changes in the hemagglutinins of Early and Recent Group subtype H3N2 influenza viruses

Virus strain^*a*^	Abbreviation	Amino acid identity at each position (H3 numbering)^*b*^											
		25	50^*c*^	57	75	137^*d*^	172^*e*^	192^*e*^	202^*f*^	222	386	452	530
A/Aichi/2/1968 (H3N2)	AI68	L	K	R	H	N	D	T	V	W	E	R	V
A/England/42/1972 (H3N2)	EN72	L	K	R	H	N	D	T	V	W	E	R	V
A/Victoria/3/1975 (H3N2)	VI75	L	K	R	H	N	D	T	V	W	E	R	V
A/Texas/1/1977 (H3N2)	TX77	L	R	R	H	Y	D	T	V	W	E	R	V
A/Bangkok/1/1979 (H3N2)	BK79	L	R	R	H	Y	G	T	V	W	E	R	V
A/Sichuan/2/1987 (H3N2)	SI87	L	R	R	H	Y	G	T	V	W	E	R	V
A/Beijing/352/1989 (H3N2)	BE89	L	R	R	H	Y	G	T	V	W	E	R	V
A/Beijing/32/1992 (H3N2)	BE92	L	R	R	H	Y	G	T	V	W	E	R	V
A/Wuhan/359/1995 (H3N2)	WU95	L	R	R	H	Y	D	T	V	W	E	R	V
A/Sydney/5/1997 (H3N2)	SY97	L	R	R	H	Y	D	T	V	W	E	R	V
A/Fujian/411/2002 (H3N2)	FU02	I	G	Q	Q	S	E	I	I	R	G	K	A
A/California/7/2004 (H3N2)	CA04	I	G	Q	Q	S	E	I	I	R	G	K	A
A/Wisconsin/67/2005 (H3N2)	WI05	I	G	Q	Q	S	E	I	I	R	G	K	A
A/Perth/16/2009 (H3N2)	PE05	I	E	Q	Q	S	E	I	I	R	G	K	A
A/Victoria/361/2011 (H3N2)	VI11	I	E	Q	Q	S	E	I	I	R	G	K	A
A/Switzerland/9715293/2013 (H3N2)	SWZ13	I	E	Q	Q	S	E	I	I	R	G	K	A
A/Hong Kong/4801/2014 (H3N2)	HK14	I	E	Q	Q	S	E	I	I	R	G	K	A
A/Singapore/INFIMH-16-0019/2016 (H3N2)	SG16	I	E	Q	Q	S	E	I	I	R	G	K	A
A/Kansas/14/2017 (H3N2)	KA17	I	E	Q	Q	F	E	I	I	R	G	K	A
A/Hong Kong/2671/2019 (H3N2)	HK19	I	E	Q	Q	S	E	I	I	R	G	K	A
A/South Australia/34/2019 (H3N2)	SA19	I	E	Q	Q	S	E	I	I	R	G	K	A

^*a*^ The Early Group comprised A/Aichi/2/1968 (H3N2) to A/Sydney/5/1997 (H3N2) inclusive. The Recent Group comprised A/Fujian/411/2002 (H3N2) to A/South Australia/34/2019 (H3N2) inclusive.

^*b*^ HA numbering is mature-form (without signal peptide).

^*c*^ Located in antigenic site C (colored green in Fig. 2).

^*d*^ Located in antigenic site A (colored red in Fig. 2).

^*e*^ Located in antigenic site B (colored blue in Fig. 2).

^*f*^ Located in antigenic site D (colored magenta in Fig. 2).

We next analyzed the predicted *N*-linked glycosylation sites on these HAs and NAs to assess their evolution over time. Glycosylation of HA, particularly on the globular head, can shield antigenic sites and influence immune evasion (17, 18, 49). At baseline, the prototype strain A/Aichi/1/1968 (AI68) carried seven glycosylation sites on HA (8NSTA, 22NGTL, 38NATE, 81NETW, 165NVTM, 285NGSI, and 483NGTY). Additional sites were acquired by 1987, and two more by 1997, reaching a peak of 11 sites in SY97 (Fig. 1E; Table 4). This was followed by modest fluctuations, with one site lost in 2005, two gained by 2011, and subsequent losses in 2013 and 2017. A single gain in 2019 restored the total to approximately 13 sites in recent viruses. Overall, HA glycosylation sites showed a dynamic pattern of accumulation and refinement, with major gains in the 1970s–1990s and a more variable profile in the post-2000 era.

**TABLE 4:** Predicted glycosylation sites on the hemagglutinins of subtype H3N2 influenza A viruses

Antigenic sites^*a*^				C^*c*^	E^*d*^	E^*d*^	A^*e*^	A^*e*^	A^*e*^	A^*e*^		D^*d*^			Total
Virus strain^*b*^	8 NSTA	22 NGTL	38 NATE	45 NSSI	63 NCTL	81 NETW	122 NESF	126 NWTG	133 NGTS	144 NNSF	165 NVTM	246 NSTG	285 NGSI	483 NGTY	
A/Aichi/2/1968 (H3N2)	+ + +	+ +	+	S	D	+	T	T	N	G	+ +	N	+ +	+ +	7
A/England/42/1972 (H3N2)	+ + +	+ +	+	S	D	+ +	N	T	N	D	+ + +	N	+ +	+	7
A/Victoria/3/1975 (H3N2)	+ + +	+ +	+	S	+ +	N	N	+	N	D	+ +	N	+ +	+	8
A/Texas/1/1977 (H3N2)	+ + +	+ +	+	S	+ +	N	N	+	N	D	+ + +	N	+ +	+	8
A/Bangkok/1/1979 (H3N2)	+ + +	+ +	+	S	+ +	N	N	+	S	D	+ + +	N	+ +	+	8
A/Sichuan/2/1987 (H3N2)	+ + +	+ +	+	S	+ +	N	N	+	S	V	+ + +	+	+ +	+	9
A/Beijing/352/1989 (H3N2)	+ + +	+ +	+	S	+ +	N	N	+	S	V	+ + +	+	+ +	+	9
A/Beijing/32/1992 (H3N2)	+ + +	+ +	+	S	+ +	N	N	+	D	V	+ + +	+	+ +	+	9
A/Wuhan/359/1995 (H3N2)	+ + +	+ +	+	S	+ +	N	N	+	D	V	+ + +	+	+ +	+	9
A/Sydney/5/1997 (H3N2)	+ + +	+ +	+	S	+ +	N	-	+	+ +	I	+ + +	+	+ +	+	11
A/Fujian/411/2002 (H3N2)	+ + +	+ +	+	S	+ +	N	-	+	+	+	+ + +	+	+ +	+	12
A/California/7/2004 (H3N2)	+ + +	+ +	+	S	+ +	N	-	+	+	+	+ + +	+	+ +	+	12
A/Wisconsin/67/2005 (H3N2)	+ + +	+ +	+	S	+ +	N	D	-	+	+	+ + +	+	+ +	+	11
A/Perth/16/2009 (H3N2)	+ + +	+ +	+	S	+ +	N	-	+	+ +	K	+ + +	+	+ +	+	11
A/Victoria/361/2011 (H3N2)	+ + +	+ +	-	+	+ +	N	-	+	+ +	-	+ + +	+	+ +	+	13
A/Switzerland/9715293/2013 (H3N2)	+ + +	+ +	+	+	+ +	N	-	N	+ +	+	+ + +	+	+ +	+	12
A/Hong Kong/4801/ 2014 (H3N2)	+ + +	+ +	+	+	+ +	N	-	+	+ +	S	+ + +	+	+ +	+	12
A/Singapore/INFIMH-16-0019/2016 (H3N2)	+ + +	+ +	+	+	+ +	N	-	+	+ +	S	+ + +	+	+ +	-	12
A/Kansas/14/2017 (H3N2)	+ + +	+ +	+	+	+ +	N	-	N	+ +	K	+ + +	+	+ +	+ +	11
A/Hong Kong/2671/ 2019 (H3N2)	+ + +	+ +	+	+	+ +	N	-	+	+ +	S	+ + +	+	+ +	-	12
A/South Australia/34/ 2019 (H3N2)	+ + +	+ +	+	+	+ +	N	-	+	+ +	S	+ + +	+	+ +	-	12

^*a*^ Single letter amino acid codes are used to indicate sequences of predicted glycosylation sites.

^*b*^ Potential glycosylation sites are those crossing the default threshold of 0.5. Single letter amino acid codes are used to indicate sequences of predicted glycosylation sites. +, potential <0.5; + +, potential <0.5 and jury agreement between nine of nine neural networks or potential < 0.75, + + + : potential < 0.75 and jury agreement. Non-glycosylated sites are indicated by; – : potential < 0.5, -- : potential < 0.5 and jury agreement, whereby all nine neural networks > 0.5, --- : potential < 0.32 and jury agreement. HA numbering is mature-form (without signal peptide).

^*c*^ Located in antigenic site C (colored green in Fig. 2).

^*d*^ Located in antigenic site D (colored magenta in Fig. 2).

^*e*^ Located in antigenic site A (colored red in Fig. 2).

In contrast, NA glycosylation site dynamics were more variable and showed no clear trend of progressive accumulation. NA initially had eight predicted sites in HK68, with two sites lost by 1972 and intermittent gains and losses over the next five decades. For example, glycosylation sites were gained in 1975, 1989, 2002 and 2005, while sites were lost in 2004, 2009, 2011, and 2017. Despite this fluctuation, the overall number of predicted NA glycosylation sites remained relatively stable, typically ranging between six and nine across decades (Fig. 1E Table 5). Together, these results reveal a gradual accumulation of glycosylation sites on HA during early decades of human circulation, followed by dynamic site turnover in recent years. In contrast, NA glycosylation sites exhibited a more stochastic pattern without a consistent directional trend.

**TABLE 5:** Predicted glycosylation sites on the neuraminidases of subtype H3N2 influenza A viruses

Virus strain^*a*^	61 NITE	69 NNTI	70 NNTI	86 NWSK	93 NITG	146 NDTT	200 NATA	234 NGTC	245 NATG	329 NDSS	367 NETS	402 NRSG	Total
A/Aichi/2/1968 (H3N2)	+ +	+	+	+	Q	+ +	-	+ + +	S	D	S	- -	8
A/England/42/1972 (H3N2)	+ +	T	+	K	K	+ +	- -	+ + +	S	N	S	- -	6
A/Victoria/3/1975 (H3N2)	+ +	T	+	+	K	+ +	- -	+ + +	S	N	S	- - -	7
A/Texas/1/1977 (H3N2)	+ +	T	+	+	K	+ +	- -	+ + +	S	N	S	- - -	7
A/Bangkok/1/1979 (H3N2)	+ +	T	+	+	K	+ +	- -	+ + +	S	N	S	- -	7
A/Sichuan/2/1987 (H3N2)	+ + +	T	+	+	K	+ +	- -	+ + +	S	N	G	-	7
A/Beijing/352/1989 (H3N2)	+ + +	T	+	+	K	+ +	+	+ + +	S	-	G	-	8
A/Beijing/32/1992 (H3N2)	+ +	T	+	+	K	+ +	+	+ + +	S	-	S	-	8
A/Wuhan/359/1995 (H3N2)	+ + +	T	+	+	K	+ +	+	+ + +	S	+	S	-	8
A/Sydney/5/1997 (H3N2)	+ + +	T	+	+	K	+ +	+	+ + +	S	+	S	-	8
A/Fujian/411/2002 (H3N2)	+ + +	T	+	+	+ +	+ +	-	+ + +	S	-	S	-	9
A/California/7/2004 (H3N2)	+ + +	T	+	+	D	+ +	-	+ + +	S	-	S	-	8
A/Wisconsin/67/2005 (H3N2)	+ + +	T	+	+	+ +	+ +	- -	+ + +	S	-	S	-	9
A/Perth/16/2009 (H3N2)	+ + +	T	+	+	D	+	-	+ + +	S	-	S	-	8
A/Victoria/361/2011 (H3N2)	+ + +	T	+	+	G	+	-	+ + +	S	T	+	D	7
A/Switzerland/9715293/2013 (H3N2)	+ + +	T	+	+	G	+	-	+ + +	-	S	+	D	8
A/Hong Kong/4801/2014 (H3N2)	+ + +	T	+	+	G	+	-	+ + +	S	-	+	D	8
A/Singapore/INFIMH-16-0019/2016 (H3N2)	+ + +	T	+	+	G	+	-	+ + +	-	+	+	D	9
A/Kansas/14/2017 (H3N2)	+ + +	T	+	+	G	+	-	+ + +	-	T	+	D	8
A/Hong Kong/2671/2019 (H3N2)	+ + +	T	+	+	G	+	-	+ + +	-	S	+	D	8
A/South Australia/34/2019 (H3N2)	+ + +	T	+	+	G	+	-	+ + +	-	S	+	D	8

^*a*^ Potential glycosylation sites are those crossing the default threshold of 0.5. Single letter amino acid codes are used to indicate sequences of predicted glycosylation sites. + : potential < 0.5, + + : potential < 0.5 and jury agreement between nine of nine neural networks or potential <0.75, + + +, potential <0.75 and jury agreement. Non-glycosylated sites are indicated by -, potential <0.5; - -, potential <0.5 and jury agreement, whereby all nine neural networks are >0.5, - - -, potential <0.32 and jury agreement.

**Fig 3:**
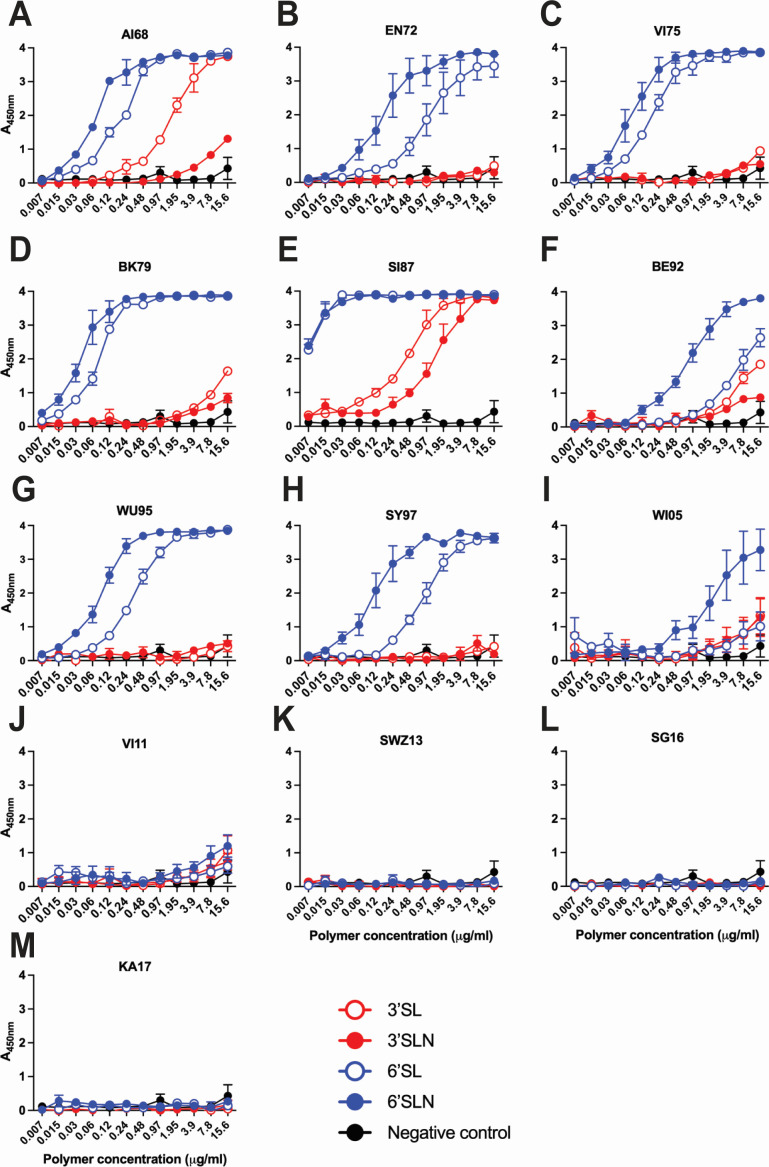
Characterization of sialic acid binding properties of subtype H3N2 influenza A viruses. Direct virus binding to the biotinylated sialic acid receptor analogs 3´SL (Neu5Acα2-3Galβ1-4Glcβ-PAA-biotin), 6´SL (Neu5Acα2-6Galβ1-4Glcβ–PAA-biotin), 3´SLN (Neu5Acα2-3Galβ1-4GlcNAc), and 6´SLN (Neu5Acα2-6Galβ1-4GlcNAcβ-PAA-biotin) was determined by solid-phase enzyme-linked receptor-binding assay. Sialic acid receptor analogs containing α2,3- or α2,6-linkages are shown in red and blue, respectively, for AI68 (**A**), EN72 (**B**), VI75 (**C**), BK79 (**D**), SI87 (**E**), BE92 (**F**), WU95 (**G**), SY97 (**H**), WI05 (**I**), VI11 (**J**), SWZ13 (**K**), SG16 (**L**), and KA17 (**M**). *N* = 3 biological replicates and *n* = 3 technical replicates were performed. Negative control data are means of no glycan control wells. Values are expressed as means ± standard error of the mean. Virus strain name abbreviations can be found in Table 1.

We next examined changes in receptor-binding preferences, which are known to evolve with prolonged human circulation (50). HA assays using red blood cells from chicken (α2,3- and α2,6-linked sialic acids) and guinea pig (predominantly α2,6-linked sialic acids) (51) revealed that the emergence of FU02 was associated with consistently higher gRBC:cRBCs HA titer ratios, indicating a stronger binding preference for α2,6-linked receptors consistent with adaptation to α2,6-rich environments (Fig. 1F). To validate this, we performed solid-phase binding assays using biotinylated sialylglycopeptides. AI68, EN72, VI75, BK79, and SI87 all showed relatively strong binding to the α2,6-linked glycans 6´SL and 6´SLN (Fig. 3A through H). However, WI05, VI11, SWZ13, and SG16, all isolated after 2002, showed markedly diminished or undetectable binding to α2,6-linked glycans (Fig. 3I through L). AI68 and SI87 were the only viruses that showed appreciable binding to α2,3-linked glycans (Fig. 3A and E). Overall, these data reveal that viruses isolated after FU02 showed a large decrease in binding affinity for the α2,6-linked glycans used in this study.

### Emergence of A/Fujian/411/2002 (H3N2) was associated with greater replication in humanized MDCK cells

**Fig 4:**
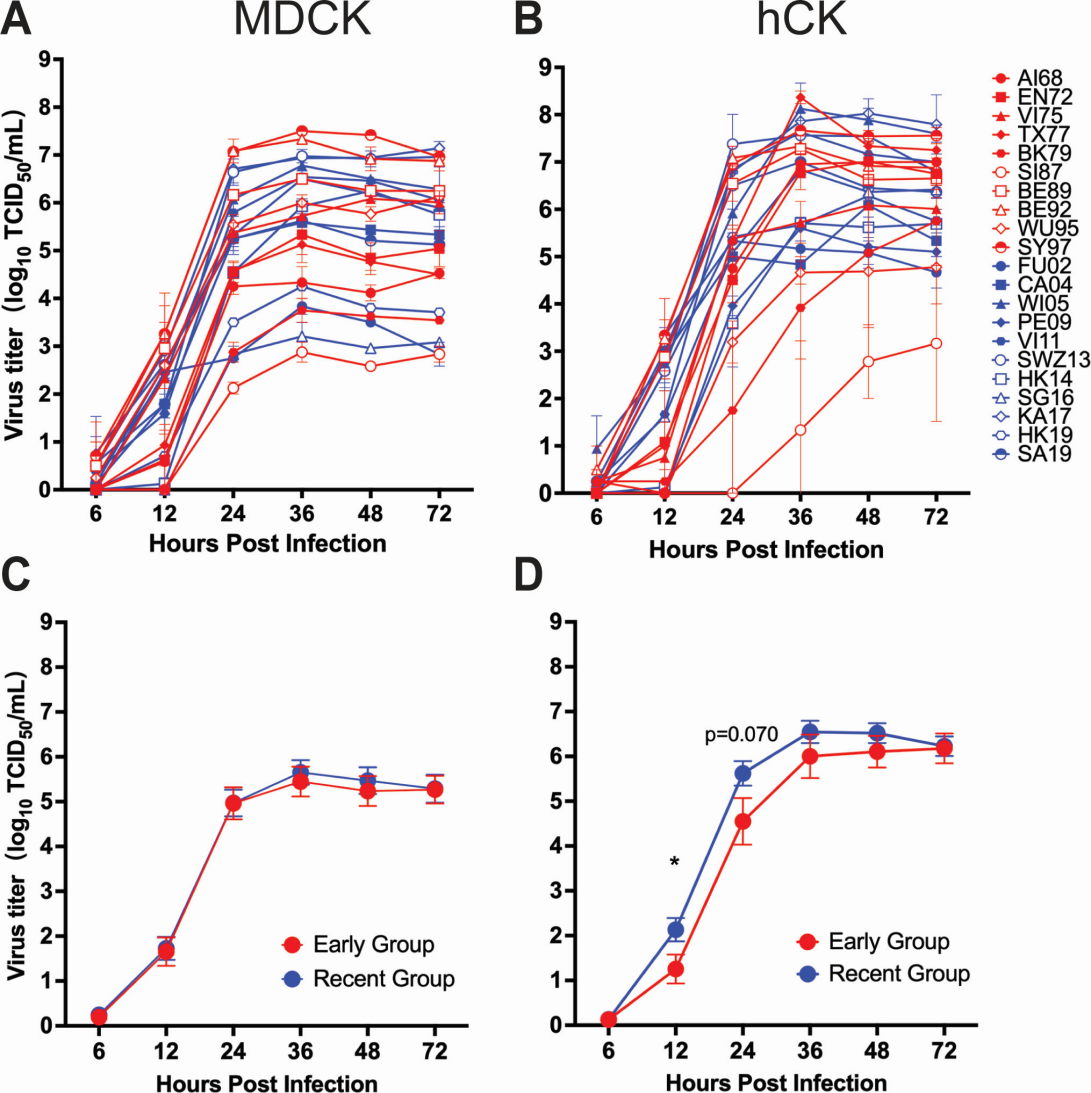
Replication of subtype H3N2 influenza A viruses in MDCK and hCK cells. Replication kinetics of subtype H3N2 influenza A viruses in MDCK (**A and C**) and hCK (**B and D**) cells. Data points in A and B represent the mean virus titer ± standard error of the mean of *n* = 3 technical replicates. Data points in C and D represent the mean virus titer ± standard error of the mean of all viruses in the designated groups. Virus strain name abbreviations can be found in Table 1. *P ≤ 0.05.

To assess functional implications of the observed shift in sialic acid specificity, we compared replication in MDCK cells and hCK cells. MDCK cells express both α2,3- and α2,6-linked sialic acids, while hCK cells have been engineered to express predominantly α2,6-linked sialic acids with extremely low expression of α2,3-linked sialic acids by CRISPR-Cas9 knockout of the genes encoding β-galactoside α-2,3 sialyltransferase (52). While all viruses replicated similarly in MDCK cells (Fig. 4A and C), recent viruses showed enhanced replication kinetics in hCK cells at 12 hours post-infection (Fig. 4B and D, *P* ≤ 0.05). These results indicate a temporal shift from mixed α2,3/α2,6 binding toward preferential, albeit weaker, α2,6 binding in more recent H3N2 viruses, likely shaped by increased HA glycosylation and human host adaptation.

### NA enzymatic activity was varied while predicted NA *N*-linked glycosylation increased over time

**Fig 5:**
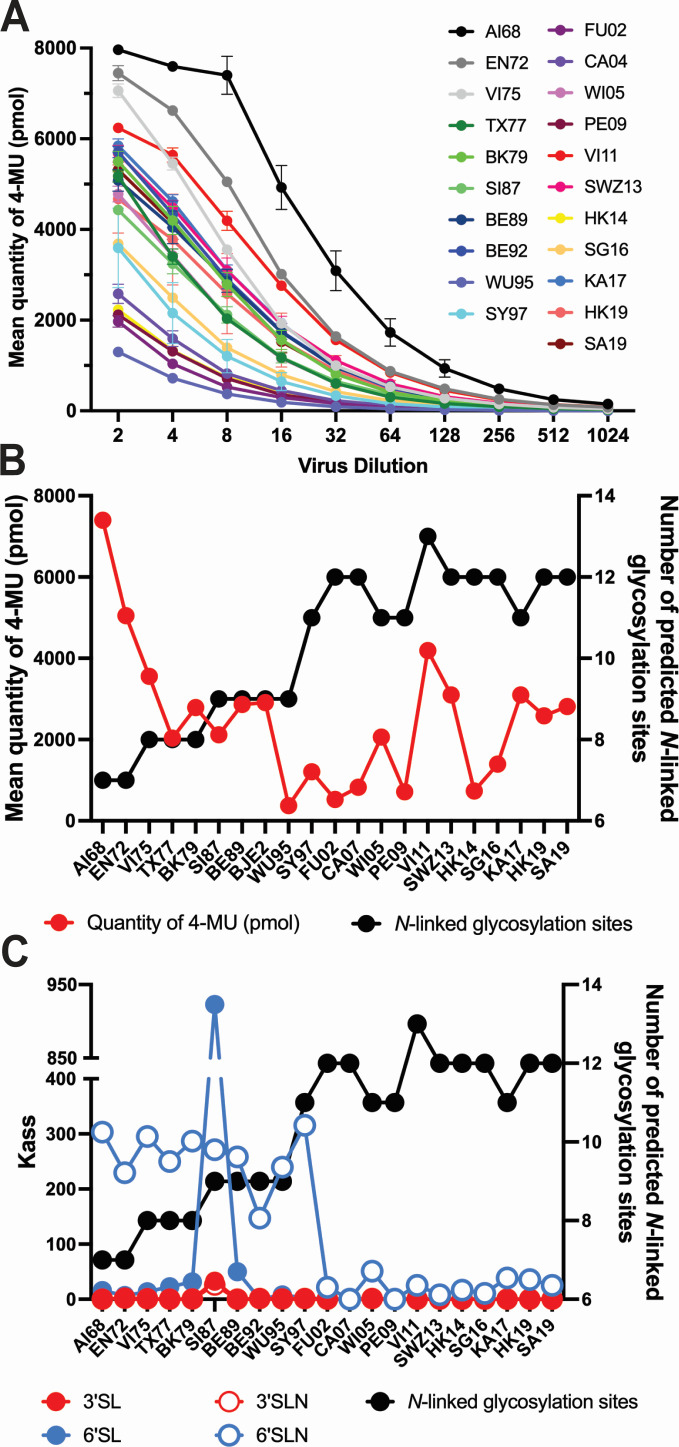
Time courses of neuraminidase enzymatic activity, predicted neuraminidase *N*-linked glycosylation sites, and hemagglutinin sialic acid affinity. Enzymatic activity of neuraminidases of subtype H3N2 influenza A viruses determined using MUNANA as a substrate (**A**). Neuraminidase enzymatic activity of subtype H3N2 influenza A viruses at 1:8 dilution and predicted *N*-linked glycosylation sites by year of isolation (**B**). Trends of hemagglutinin affinity for 3´SL (Neu5Acα2-3Galβ1-4Glcβ-PAA-biotin), 3´SLN (Neu5Acα2-3Galβ1-4GlcNAc), 6´SL (Neu5Acα2-6Galβ1-4Glcβ–PAA-biotin), and 6´SLN (Neu5Acα2-6Galβ1-4GlcNAcβ-PAA-biotin) and predicted *N*-linked glycosylation sites of subtype H3N2 influenza A viruses by year of isolation. Affinity is expressed as apparent association constants (*K*_ass_) (**C**). Virus strain name abbreviations can be found in Table 1.

NA enzymatic activity is an important component of the influenza virus lifecycle and is critical for HA-NA functional balance (53–55). As such, we studied the NA activities of these viruses using the fluorogenic substrate 2´-(4-methylumbelliferyl)-α-_D_-*N*-acetylneuraminic acid (MUNANA). AI68 demonstrated the highest NA activity, with activities subsequently decreasing until TX77, then plateauing until BE92, after which activities were more variable but generally lower than BE92, with the exception of VI11 and SWZ13 (Fig. 5A and B).

Predicted NA *N*-linked glycosylation sites showed an increasing trend, from 7 on AI68 NA to 9 on SI87 to 12 on FU02 before plateauing, varying from 11 to 13 for the remaining viruses (Fig. 5B). Overall, there was no correlation between the number of predicted NA *N*-linked glycosylation sites and NA enzymatic activity (*R*^2^ = 0.2331), but NA enzymatic activity showed an overall decreasing trend over time (Fig. 5B).

### Correlation between predicted NA *N*-linked glycosylation sites and HA affinity for 6´SLN sialic acids

We next studied the time course of HA affinity for α2,3- and α2,6-linked sialic acids. Affinities for 6´SL sialic acids were relatively stable, with the exception of SI87, which showed a much greater affinity compared to other viruses (Fig. 5C). Affinities for 3´SL and 3´SLN sialic acids were lower compared to 6´ sialic acids until FU02, whereby affinities for 6´ sialic acids decreased approximately 10-fold from 259.8 ± 15.4 to 26.4 ± 5.2 (Fig. 5C). Overall, there was also a negative correlation between the number of predicted NA *N*-linked glycosylation sites and HA affinity for 6´SLN sialic acids (*R*^2^ = 0.719).

### Attenuated pathogenicity of more recent strains in the mouse model

**Fig 6:**
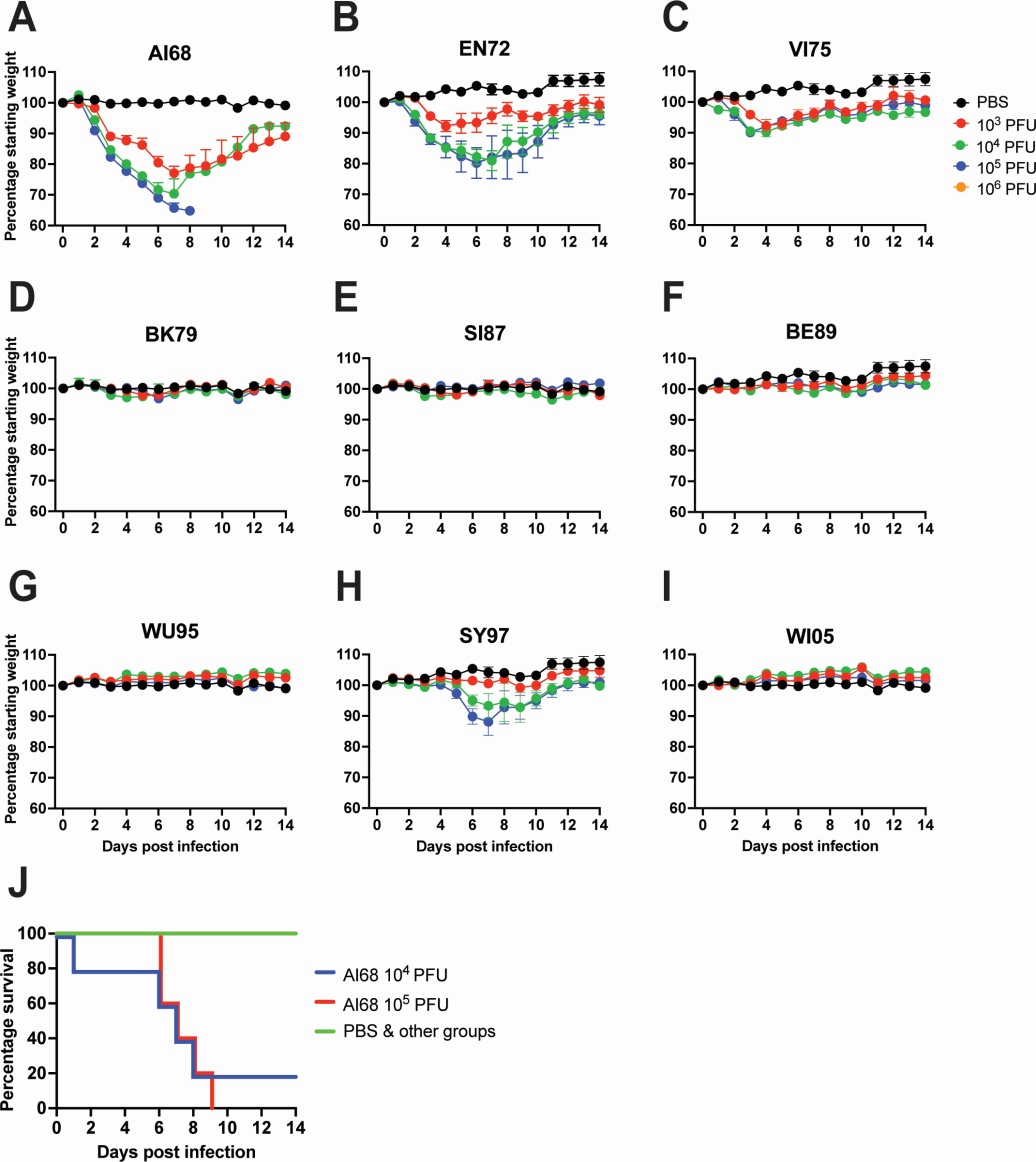
Mammalian pathogenicity of subtype H3N2 influenza A viruses in the mouse model. Mice were inoculated intranasally at 10^3^, 10^4^, or 10^5^ plaque-forming units (red, green, and blue, respectively). Percentage mean initial body weight for mice inoculated with AI68 (**A**), EN72 (**B**), VI75 (**C**), BK79 (**D**), SI87 (**E**), BE89 (**F**), WU95 (**G**), SY97 (**H**), and WI05 (**I**) are shown. Data points represent the mean percentage starting body weight ± standard error of the mean of *n* = 5 mice per group. PBS group data shown in A–I are from the same mice. Survival rates of inoculated mice are shown in (**J**).

**Fig 7:**
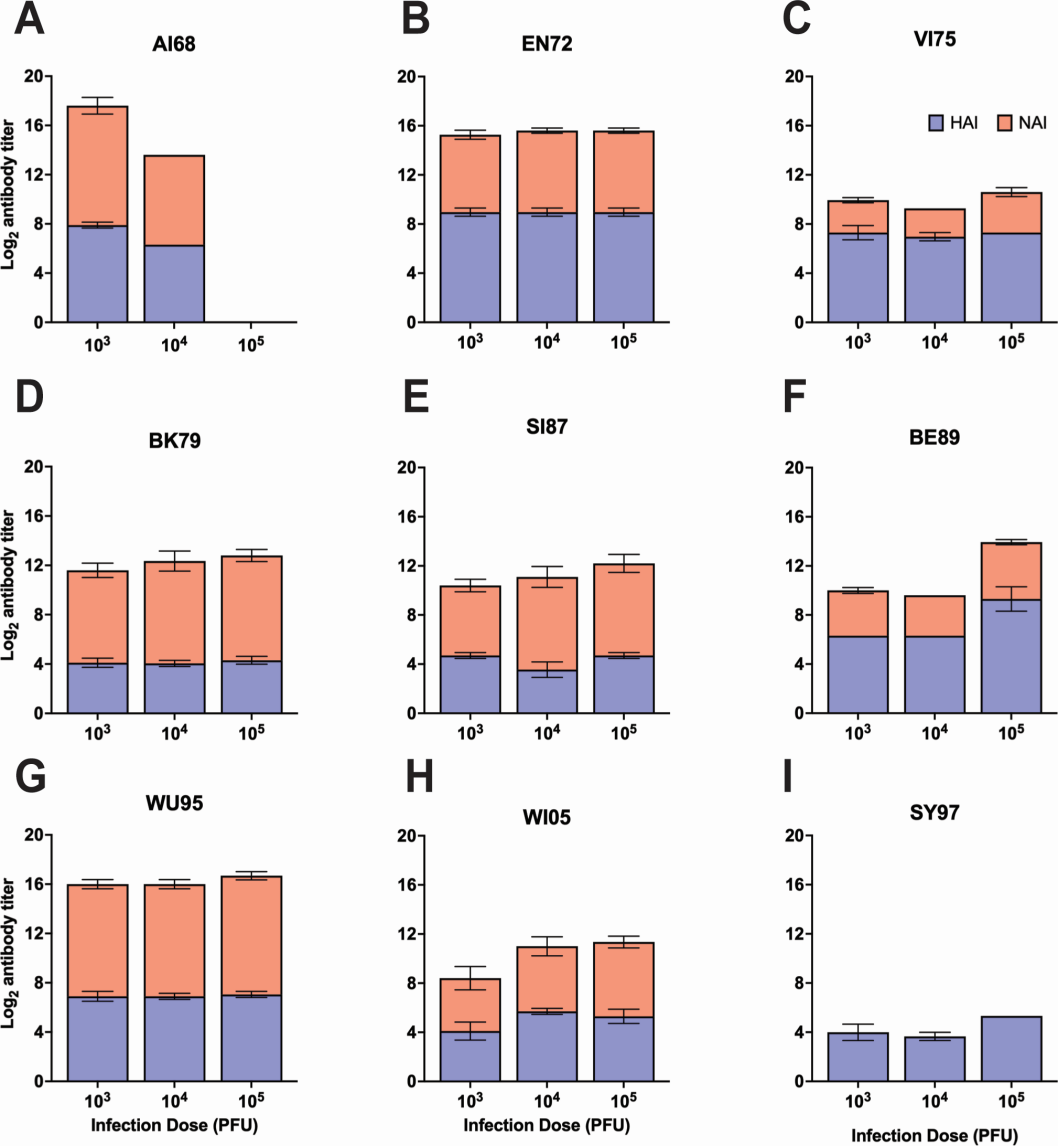
Serum antibody responses to hemagglutinin and neuraminidase elicited by subtype H3N2 influenza A viruses in the mouse model. Log_2_ mean HAI and NAI antibody titers (blue and orange, respectively) measured in serum collected at 28 days post-inoculation with AI68 (**A**), EN72 (**B**), VI75 (**C**), BK79 (**D**), SI87 (**E**), BE89 (**F**), WU95 (**G**), WI05 (**H**), and SY97 (**I**) (*n* = 5 mice per group). Virus strain name abbreviations can be found in Table 1. Limit of detection for these assays was 1:10 (log_2_ 3.3). Graphs show the mean and the ± standard error of the mean. Serum samples were not collected from the group inoculated with 10^5^ plaque-forming units of AI68 as all mice reached humane endpoints prior to 28 days post-inoculation. NAI titers against SY97 were not determined due to lack of serum (**I**).

We next used the mouse model to determine the impact of these adaptations on mammalian pathogenicity using 9 H3N2 viruses. AI68 and EN72 were the most pathogenic, with all titers causing weight loss. At 10^5^ PFU, mean body weights of mice inoculated with AI68 or EN72 dropped to a minimum of 73.76 ± 0.41% and 80.20 ± 4.99% of starting body weights, respectively (Fig. 6A and B). VI75 and SY97 were also pathogenic, with all titers of VI75 causing weight loss and 10^4^ and 10^5^ PFU of SY97 causing weight loss. At 10^5^ PFU, mean body weights of mice inoculated with VI75 or SY97 dropped to 90.11 ± 0.79% and 88.11 ± 4.42% of starting body weights, respectively (Fig. 6C and H). The other IAVs tested, namely the Early Group BK79, SI87, BE92, and WU95 and the Recent Group WI05, did not cause any significant mean weight loss (Fig. 6D through G and I). AI68 was the only virus to cause mice to reach humane endpoints, with 0% and 20% survival evident in mice inoculated with 10^5^ and 10^4^ PFU, respectively (Fig. 6J). We next used mouse serum collected 28 days post-inoculation to determine hemagglutination inhibition (HAI) and NAI antibody titers. We detected HAI and NAI titers in all mice, indicating that mice were infected and mounted immune responses (Fig. 7). Overall, these data indicate that, apart from SY97, the earlier H3N2 viruses were the most pathogenic and that all viruses were immunogenic.

## DISCUSSION

The continuing evolution of H3N2 viruses since their introduction into humans in 1968 has revealed insights into several virus-host interactions driving the selective pressures on these IAVs. Our goal was to use multiple approaches to study the mammalian adaptation of the HAs and NAs of H3N2 IAVs selected to represent antigenic clusters from 1968 to 2003 and subsequent vaccine strains from 2004 to 2019. Our findings highlight divergent evolutionary strategies in HA and NA, whereby HA accumulates glycosylation for immune evasion and receptor adaptation, while NA maintains enzymatic function despite stochastic glycosylation changes. This would agree with previous studies that have identified discordant antigenic drift between HA and NA, particularly between WU95 and SY97, whereby a major step in HA antigenic drift was observed with almost no changes in NA (56–58).

The post-2002 shift to α2,6-linked sialic acid binding, coupled with reduced pathogenicity in mammalian models, underscores H3N2 adaptation to human circulation without affecting immunogenicity, which is a critical consideration for vaccine design. More recently, H3N2 NAs appear to be undergoing incremental changes. Mutations have accumulated in exposed positions on the globular head domain, many of them near the active site and the central Ca^2+^ binding site that is critical for NA activity in the 2020–2021 vaccine strains, compared to HK19 (59, 60). However, these NAs were still antigenically similar to HK19 (59).

Our genetic data revealed that the transition from SY97 to FU02 delineated the Early and Recent Groups, as observed previously (31, 32, 41). The emergence of FU02 marked a departure from the A/Moscow/10/1999 (H3N2)-like or antigenically equivalent A/Panama/2007/1999 (H3N2)-like IAVs. This was evident in the reduced VE observed when A/Panama/2007/1999 (H3N2) was used as the vaccine strain for the 2003–2004 influenza season, which led to the selection of the FU02-like A/Wyoming/03/2003 (H3N2) for the 2004–2005 influenza season (61, 62). A/Wyoming/03/2003 (H3N2) differed at 16 amino acids in HA1 compared to A/Panama/2007/1999 (H3N2), but introducing H155T and Q156H into A/Panama/2007/1999 (H3N2) was sufficient to make this virus antigenically equivalent to A/Wyoming/03/2003 (H3N2) (34). FU02-like IAVs resulted in epidemics in China, Japan, and South Korea and became the dominant IAVs in Asia (6, 31, 33). Sequence comparisons revealed 12 amino acids differentiating the Early and Recent Groups, which did not include positions 155 and 156.

Our receptor-binding data revealed a large decrease in binding affinity for α2,6-linked sialic acids in IAVs isolated after FU02, in agreement with other studies. A large decrease in binding affinity for α2,6-sialyl lactosamine in H3N2 viruses isolated after 2001 and reductions in binding specificities for α2,6-linked sialic acids in H3N2 viruses isolated in 2006, 2008, and 2010 compared to an isolate from 1992 have been observed (13, 20). Binding specificity changes have been observed, with the dual specificity of AI68 for α2,3- and α2,6-linked sialic acids changing to a predominant α2,6-linked sialic acid specificity that then declined after 1995 (50). Similarly, compared to an earlier H3N2 virus, more recent H3N2 viruses showed much more restricted binding to a subset of glycans with α2,6-linkages (63). Preferences for glycans with longer, linear chains have also been observed, with the longer glycans postulated to engage the binding sites of two HA monomers in the same trimer, increasing binding avidity (15, 16). Here, we also observed binding to the longer 6´SLN sialylglycopeptide compared to the shorter 6´SL sialylglycopeptide. Interestingly, our data also indicate that these changes in binding largely differentiated the Early and Recent Group viruses, suggesting an evolutionary event differentiating SY97 and FU02.

Studies of antigenic evolution describe a trend of incremental increases in *N*-linked glycosylation sites on the globular head domain of HA. This has the effect of shielding the RBS from antibodies and contributing to poor VE, despite vaccine strains being well matched to circulating strains (17, 18). This pattern was also evident in the IAVs studied here, with increasing numbers of predicted HA glycosylation sites until SY97, after which the numbers remained relatively stable, although we did not study the impact of increasing glycosylation sites on VE and the immunogenicity of these viruses. Interestingly, this also corresponded to the transition between the Early and Recent Groups. However, changes in binding preferences and glycosylation do not necessarily correlate with changes in infectivity or replication *in vitro* or pathogenicity in the mouse model (64). We also noted this here, with Recent Group viruses showing greater replication in hCK cells but reduced pathogenicity in the mouse model. This may reflect adaptation to the human host rather than mammalian hosts in general.

The pathogenicity of H3N2 viruses has been observed to decrease with continued human circulation (65, 66). Here, we saw a similar trend, with AI68 and IAVs isolated in the 1970s causing symptomatic infections in mice, and AI68 being the only IAV to cause mice to reach humane endpoints. IAVs isolated after the 1970s did not lead to any observable symptoms in mice apart from the higher doses of SY97. Changes in pathogenicity did not appear to correlate with changes in genetic relatedness, receptor-binding specificity, or replication of these IAVs, with Early Group IAVs isolated from 1979 to 1995 inclusive and all Recent Group IAVs eliciting no detectable symptoms in mice. The reasons behind this, and the pathogenicity of SY97, are unclear based on the data obtained in this study but may warrant further investigation.

We used the mouse model to study mammalian pathogenicity, which is widely used but has limitations in the context of human pathogenicity. There are notable differences between the human and murine respiratory tracts in the context of influenza virus infection. The human and murine respiratory tracts show similar expression of α2,3-linked sialic acids but not α2,6-linked sialic acids, which are present in the human but not the mouse respiratory tract (67, 68). Human H3N2 and H1N1 IAVs, which are generally adapted to bind to α2,6-linked sialic acids, show binding to human trachea, bronchus, bronchioles, and alveoli but not to mouse trachea, bronchus, and bronchioles, and only rare binding to mouse alveoli (69). The ferret model represents a better approximation of the human respiratory tract, showing predominant influenza virus infection of the upper respiratory tract and expression of α2,6-linked sialic acids (70). However, the costs and husbandry of ferrets are prohibitive for such a study.

There were some limitations to this study. It should be noted that these IAVs were “6 + 2” reassortants, meaning they contained H3N2 HA and NA genes with the internal genes of PR8. As such, this study focused on the IAV surface glycoproteins and did not study other possible determinants of mammalian fitness related to the internal genes, which have been described (71). Furthermore, the use of 6 + 2 viruses meant that the binding specificities could not conclusively be attributed to HA and may be impacted by NA sialic acid binding, as observed previously (72, 73). As this study was focused on binding preferences for α2,3- or α2,6-linked sialic acids, we used a limited number of glycans, which is restrictive to the depth of interpretation of binding specificities. Further studies using larger numbers of glycans would yield more information.

Where possible, the HA and NA sequences in this study were obtained from cell-propagated viruses. However, this was not possible for some older viruses, for which HA and NA sequences were only available from egg-propagated viruses (Table 1). While this may have implications for sialic acid binding preferences, we did not find obvious differences between viruses in the Early Group that were generated using sequences derived from egg- or cell-passaged viruses. IAVs can accumulate mutations during egg passage, particularly in the HA receptor-binding site, to facilitate binding to α2,3-linked sialic acids (74). These mutations may also affect antigenicity as the receptor-binding site overlaps with HA antigenic sites (75). This phenomenon has been observed particularly for egg-grown H3N2 vaccine strains, such as G186V and L194P, which affect antigenicity, and T160K, which causes the loss of a glycosylation site (76, 77).

While we observed that SY97 and FU02 delineated the Early and Recent Groups, our study did not include IAVs isolated between 1998 and 2001. Further studies of these IAVs may give more insights into this transition. It should also be noted that MUNANA is a relatively small substrate for NA compared to glycans, meaning that NA activity measured by MUNANA should be taken as a proxy for NA activity using glycans as a substrate. Furthermore, our glycosylation data were also derived from prediction and not experimentally validated.

In summary, we found that the continuous genetic evolution of H3N2 IAV HA and NA since their 1968 introduction into humans showed a surge of mammalian adaptation between 1997 and 2002, leading to the emergence of FU02 viruses and subsequent viruses with more hallmarks of mammalian adaptation and reduced pathogenicity in mammalian models. The post-2002 shift to α2,6-linked sialic acid binding, coupled with reduced pathogenicity in mammalian models, underscores H3N2 virus adaptation to human circulation without affecting immunogenicity, which is a critical consideration for vaccine design. This study has, therefore, yielded new findings on the evolutionary time course of H3N2 viruses in humans.

## Data Availability

The data sets generated in this study are available on reasonable request.
